# Telomerase-mediated immortalization preserves the anti-inflammatory activity of dental pulp stem cell extracellular vesicles

**DOI:** 10.3389/fimmu.2026.1833834

**Published:** 2026-06-10

**Authors:** Sadiq Umar, Yu Lu, Sherly Makar, Koushik Debnath, Wang Li, Sreeparna Chakraborty, Jalees Rehman, Sriram Ravindran

**Affiliations:** 1 Department of Oral Biology, University of Illinois, Chicago, IL, United States; 2 Department of Biochemistry and Molecular Genetics, University of Illinois, Chicago, IL, United States

**Keywords:** acute lung injury, dental pulp stem cells, extracellular vesicles, hTERT, immortalized DPSC cells, inflammation

## Abstract

**Background:**

Extracellular vesicles (EVs) derived from dental pulp stem cells (DPSCs) exhibit immunomodulatory activity in multiple inflammatory models. However, the limited replicative lifespan of primary DPSCs and passage-dependent functional variability restrict scalable and reproducible EV production. Approaches that stabilize EV bioactivity during extended cell expansion are therefore needed.

**Methods:**

Human primary DPSCs were immortalized via expression of human telomerase reverse transcriptase (hTERT) to mitigate senescence-associated limitations. EVs isolated from naïve and hTERT-immortalized DPSCs were characterized for size distribution, morphology, marker expression, and cellular endocytosis. Immunomodulatory activity of EVs isolated from multiple passages of immortalized DPSCs was evaluated *in vitro* on lipopolysaccharide (LPS)/interferon-γ–activated primary mouse bone marrow–derived macrophages in comparison with primary DPSC EVs. *In vivo* efficacy was assessed in a murine model of LPS-induced acute lung injury.

**Results:**

hTERT expression increased telomerase levels without altering the multi-lineage differentiation potential of the cells. No significant changes in EV size, morphology, or expression of canonical EV markers was observed. EV uptake by macrophages was unchanged between naïve and immortalized DPSC-derived EVs. Both EV sources similarly suppressed pro-inflammatory cytokine expression, including TNF-α, IL-1β, and IL-6, in inflamed primary macrophages. Notably, EVs derived from hTERT-immortalized DPSCs retained immunomodulatory activity through at least passage 15. *In vivo*, EVs from naïve and immortalized DPSCs comparably reduced pulmonary edema, inflammatory gene expression, and neutrophil accumulation following LPS challenge. Similar preservation of EV activity was observed using EVs derived from immortalized human bone marrow derived mesenchymal stem cells.

**Conclusion:**

hTERT-mediated immortalization of parent DPSCs retains the immunomodulatory function of derivative EVs during extended culture without altering key physicochemical EV characteristics. Immortalized DPSCs represent a reproducible source of EVs with consistent anti-inflammatory activity, supporting their use as a translationally-relevant platform for EV-based immunomodulatory applications.

## Introduction

Dental pulp stem cells (DPSCs) have emerged as a highly promising cell source in regenerative medicine due to their minimally invasive isolation approach, multilineage differentiation potential, and potent paracrine activity (1–4). Extensive preclinical studies have demonstrated the therapeutic utility of DPSCs in tissue repair and regeneration across multiple organ systems, including bone, neural tissue, liver, cornea, myocardial infraction and cartilage (5–13). Increasing evidence suggests that many of these regenerative and immunomodulatory effects are mediated not by direct cell engraftment, but by extracellular vesicles (EVs) secreted by DPSCs, which serve as critical conveyors of bioactive proteins, lipids, and regulatory RNAs (11, 14–18).

Despite their therapeutic promise, the clinical translation of DPSCs and DPSC-derived EVs is hindered by several fundamental limitations. Primary DPSCs exhibit a finite replicative lifespan *in vitro*, undergoing replicative senescence after a limited number of passages, typically beyond passage 4–5. This senescence is accompanied by reduced proliferative capacity, altered secretory profiles, and diminished therapeutic efficacy (19–22). Importantly, prolonged *in vitro* expansion not only affects the cells themselves but also profoundly impacts the biological activity of their secreted EVs. Consequently, early-passage DPSC-EVs are often recommended for therapeutic applications, creating a significant bottleneck for scalable and standardized EV production (23). Moreover, EV cargo composition is highly dependent on donor cell type, passage number, and cellular state, with distinct miRNA signatures contributing to variability in therapeutic outcomes across studies (24).

At the molecular level, replicative senescence is closely linked to progressive telomere shortening. Telomeres are repetitive nucleotide sequences that cap chromosome ends and preserve genomic integrity during cell division (25, 26). In somatic cells, telomeres shorten with each replication cycle, and once a critical length is reached, cells enter irreversible growth arrest and senescence. Telomere attrition is therefore a key driver of cellular aging and functional decline and has been strongly associated with age-related diseases and reduced tissue regenerative capacity (27, 28). Conversely, maintenance of telomere length is a hallmark of long-lived and highly proliferative cells. Human telomerase reverse transcriptase (hTERT), the catalytic subunit of telomerase, counteracts telomere shortening and has been widely used to extend the replicative lifespan of primary cells (29–31). While this approach immortalizes the cells, it also renders them unusable for cellular transplantations in regenerative medicine owing to high risks of oncogenic outcomes. However, the secreted EVs from these immortalized cells do not carry the plasmid DNA and may be a good source for EVs with replicable properties should EV properties remain unaltered after immortalization. The LPS-induced acute lung injury model was selected as a well-established system to evaluate macrophage-driven innate immune responses and neutrophil recruitment, enabling robust assessment of EV-mediated immunomodulation.

In this study, we employed hTERT-mediated immortalization of DPSCs as a strategy to overcome senescence-associated limitations and enable sustained EV production. We systematically characterized EVs derived from naïve and hTERT-immortalized DPSCs across multiple passages, focusing on their physicochemical properties, molecular cargo, and immunomodulatory function. Using both *in vitro* macrophage polarization assays and *in vivo* murine acute lung injury model, we evaluated whether hTERT immortalization alters EV uptake, anti-inflammatory efficacy, or innate immune regulatory capacity. Our findings demonstrate that hTERT-mediated immortalization preserves EV composition and function, enabling long-term production of bioactive DPSC-derived EVs with sustained anti-inflammatory activity, thereby providing a scalable and functionally stable EV source for therapeutic applications.

## Results

### Extracellular vesicles from DPSCs and hTERT-DPSCs exhibit comparable physicochemical properties

We and others have previously demonstrated that EVs derived from dental pulp stem cells (DPSCs) exhibit robust anti-inflammatory activity (11, 17). DPSCs were immortalized by ectopic expression of human telomerase reverse transcriptase (hTERT), and the resulting cells were systematically compared with naïve DPSCs for both physicochemical EV characteristics and functional properties.

**Figure 1:**
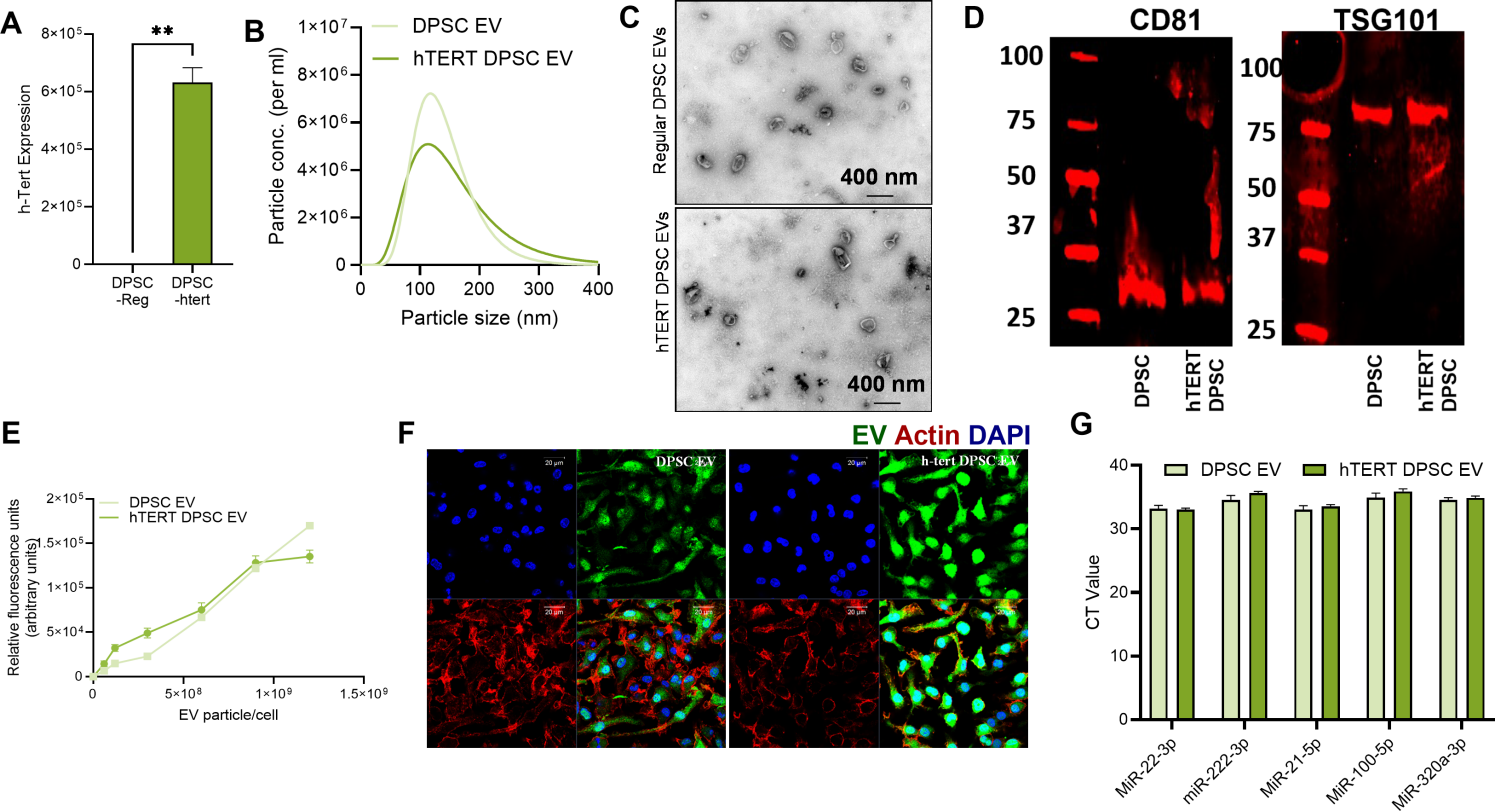
Characterization and uptake of extracellular vesicles derived from naïve and hTERT-immortalized DPSCs. Extracellular vesicles (EVs) were isolated from conditioned media of naïve and hTERT-immortalized dental pulp stem cells (DPSCs). **(A)** Relative hTERT mRNA expression in naïve and immortalized DPSCs assessed by qRT-PCR. **(B)** Representative nanoparticle tracking analysis (NTA) profiles showing EV size distribution. **(C)** Transmission electron microscopy (TEM) images demonstrating typical vesicular morphology (scale bar indicated). **(D)** Immunoblot analysis of EV markers CD81 and TSG101. **(E)** Quantitative uptake of fluorescently labeled EVs by mouse bone marrow–derived macrophages (mBMMΦs) measured after 2 h incubation. **(F)** Representative confocal microscopy images showing intracellular localization of EVs (green) within macrophages, with actin cytoskeleton (red) and nuclei (blue). Data are representative of at least three independent experiments. **(G)** miRs expression in EVs isolated from naïve and hTERT-DPSC. Data are presented as mean ± SEM (n = 3). Statistical significance was determined by one-way ANOVA followed by Šídák’s multiple-comparison test. **p < 0.01.

Immortalized DPSCs displayed a marked and significant increase in hTERT mRNA expression compared with naïve DPSCs, confirming successful cellular immortalization (**Figure 1A**). We next evaluated whether hTERT expression altered EV biophysical properties. Nanoparticle tracking analysis (NTA) revealed no significant differences in EV particle size distribution between naïve and hTERT-DPSC-derived EVs (**Figure 1B**). Transmission electron microscopy (TEM) further demonstrated comparable vesicular morphology between the two EV populations (**Figure 1C**). Consistent with these findings, immunoblot analysis showed similar expression levels EV markers in EVs isolated from naïve and hTERT-immortalized DPSCs (**Figure 1D**). To assess whether immortalization affected EV uptake, both quantitative and qualitative endocytosis assays were performed using mouse bone marrow–derived macrophages (mBMMΦs). As shown in **Figures 1E, F**, EVs derived from naïve and hTERT-DPSCs exhibited comparable endocytic uptake by macrophages, indicating preserved cellular internalization capacity following immortalization. Finally, qRTPCR analysis of top five highly expressed EV miRNA cargo revealed no significant alterations in miRNA levels between naïve and hTERT-DPSC EVs (**Figure 1G**). These results are presented as raw Ct values as no housekeeping miRNAs are available for EVs. Similar results were observed in human bone marrow derived MSCs (BM-MSCs, **Supplementary Figure 1**).

Collectively, these results demonstrate that hTERT-mediated immortalization of DPSCs does not alter EV physicochemical characteristics, uptake efficiency, or miRNA cargo, supporting the use of hTERT-DPSC-derived EVs as a stable and functionally preserved EV source.

### hTERT immortalization does not alter the multilineage differentiation potential of DPSCs

To determine whether hTERT-mediated immortalization affects the intrinsic differentiation capacity of dental pulp stem cells, we compared the osteogenic, chondrogenic, and adipogenic differentiation potential of naïve and hTERT-immortalized DPSCs under lineage-specific induction conditions. Cells were cultured in osteogenic, chondrogenic, or adipogenic differentiation media, and lineage commitment was assessed using both histological staining and gene expression analyses.

**Figure 2:**
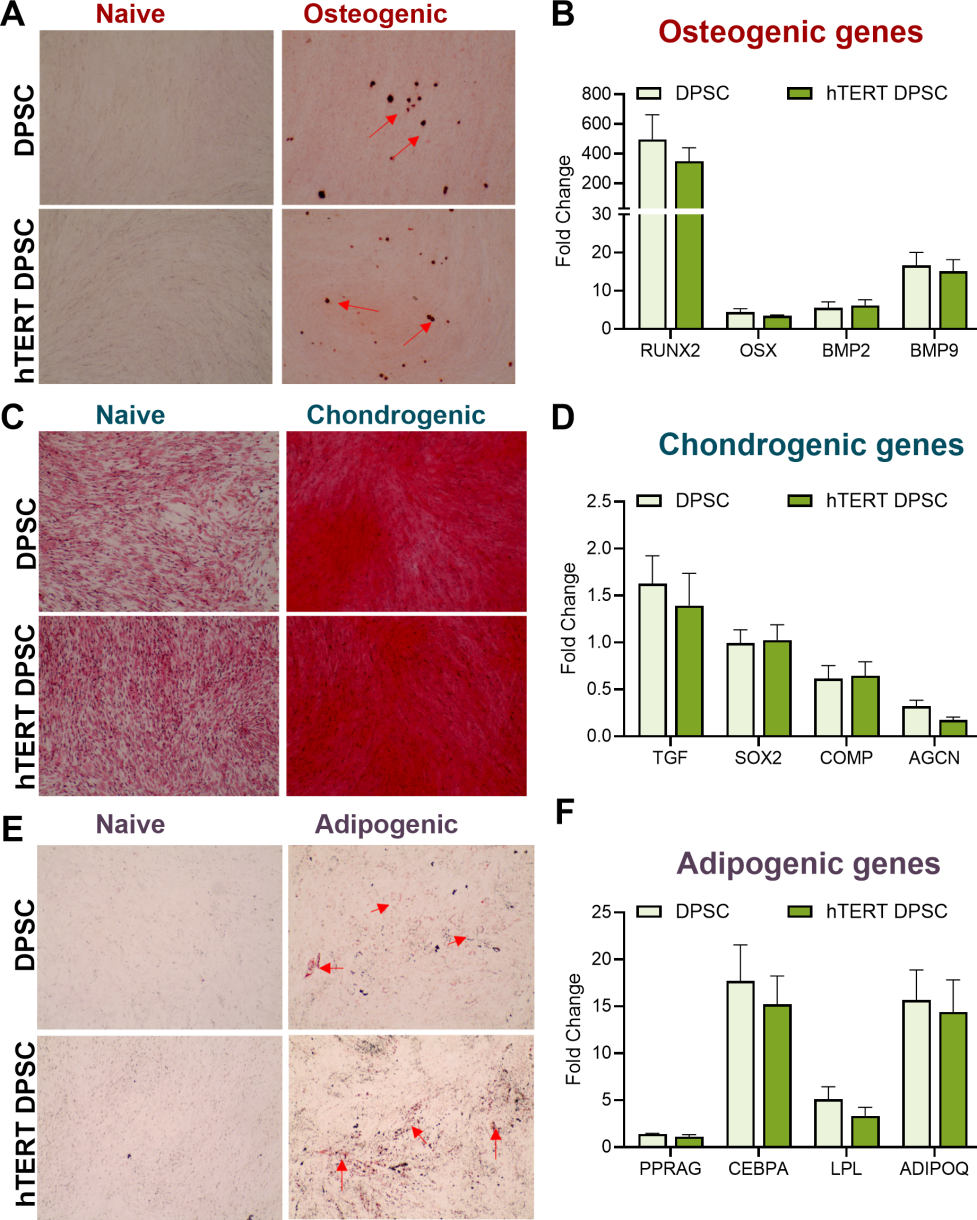
hTERT-mediated immortalization does not alter multilineage differentiation potential of DPSCs. Naïve and hTERT-immortalized DPSCs were cultured under lineage-specific differentiation conditions. **(A)** Osteogenic differentiation assessed by Alizarin Red S staining of mineralized matrix. **(C)** Chondrogenic differentiation evaluated by Safranin staining of proteoglycan-rich extracellular matrix. **(E)** Adipogenic differentiation visualized by Oil Red O staining of intracellular lipid droplets. **(B, D, F)** Quantitative gene expression analysis of lineage-specific osteogenic, chondrogenic, and adipogenic markers by qRT-PCR. Representative images and quantitative data indicate comparable multilineage differentiation capacity between naïve and immortalized DPSCs. Data are presented as mean ± SEM (n = 3).

Under lineage-specific differentiation conditions, both naïve and hTERT-immortalized DPSCs exhibited comparable multilineage differentiation capacity. Osteogenic induction resulted in robust mineralized matrix deposition in both cell populations, as demonstrated by similar Alizarin Red S staining intensity (**Figure 2A**). Likewise, chondrogenic differentiation led to equivalent production of proteoglycan-rich extracellular matrix in naïve and hTERT-DPSCs, as evidenced by comparable Safranin staining (**Figure 2C**). Adipogenic induction also produced similar intracellular lipid accumulation in both groups, visualized by Oil Red O staining, indicating preserved adipogenic differentiation following hTERT-mediated immortalization (**Figure 2E**).

Consistent with these findings, gene expression analyses revealed no significant differences in lineage-specific marker induction between naïve and hTERT-DPSCs. Key osteogenic, chondrogenic and adipogenic genes were similarly upregulated in both cell types following differentiation (**Figures 2B, D, F**). Together, these results demonstrate that hTERT-mediated immortalization does not interfere with the multilineage differentiation capacity of DPSCs. hTERT-DPSCs retain osteogenic, chondrogenic, and adipogenic differentiation potential comparable to naïve DPSCs, supporting their use as a functionally preserved and expandable cell source for EV production and regenerative applications.

### Comparative analysis demonstrates that hTERT immortalization does not affect the anti-inflammatory function of DPSC-derived EVs

**Figure 3:**
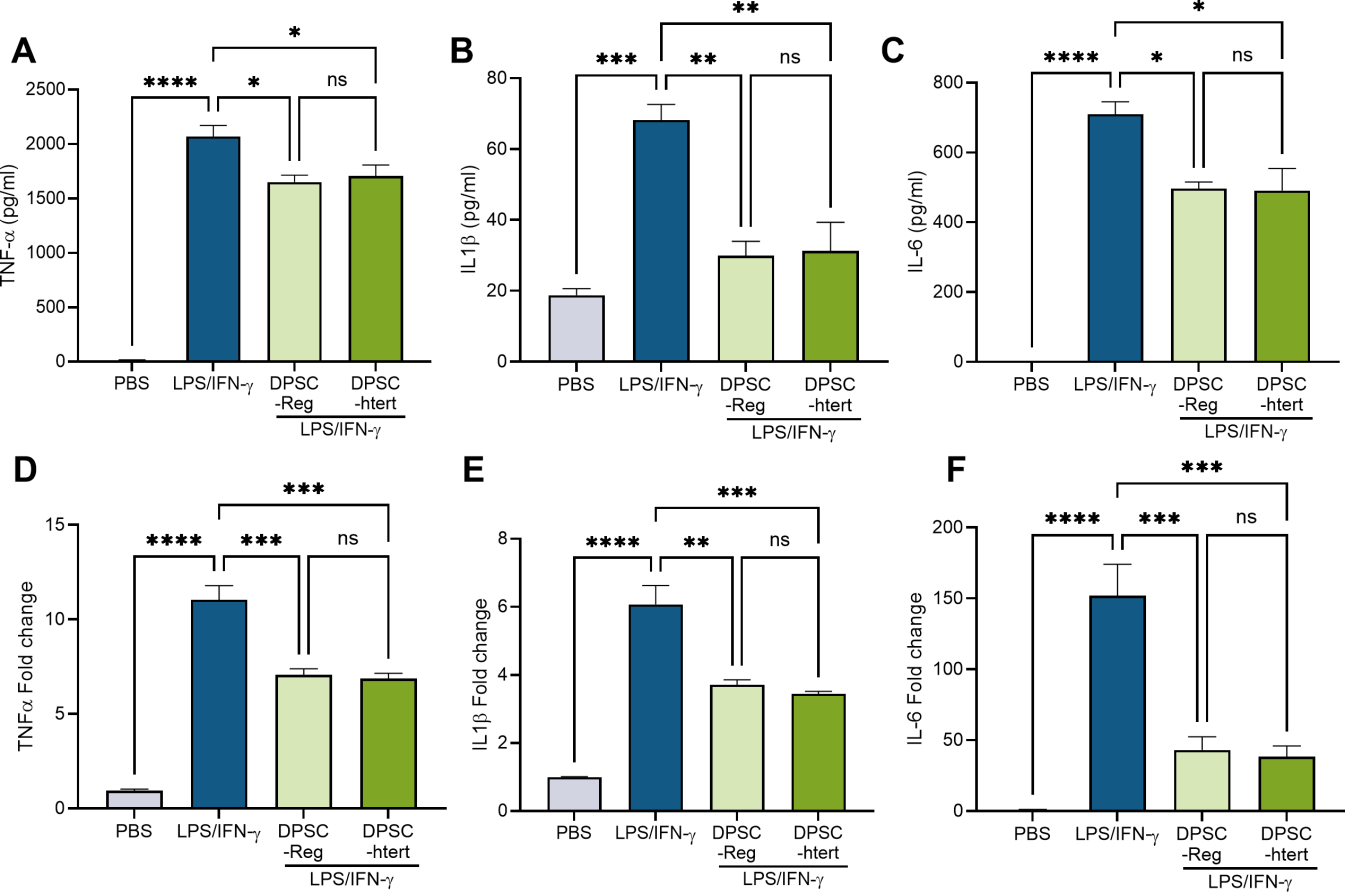
EVs from naïve and hTERT-immortalized DPSCs similarly suppress inflammatory activation of macrophages *in vitro.* Primary mouse bone marrow–derived macrophages (mBMMΦs) were activated with LPS and IFN-γ and treated with EVs derived from naïve or hTERT-immortalized DPSCs. **(A–C)** Secreted cytokine levels (TNF-α, IL-1β, and IL-6) measured in conditioned media by ELISA. **(D–F)** Relative mRNA expression of pro-inflammatory cytokines TNF-α, IL-1β, and IL-6 assessed by qRT-PCR. Data are presented as mean ± SEM (n = 3). Statistical significance was determined by one-way ANOVA followed by Šídák’s multiple-comparison test. ns- not significant, *p < 0.05, **p < 0.01, ***p < 0.001, ****p < 0.0001.

We next evaluated whether hTERT-mediated immortalization alters the anti-inflammatory activity of DPSC-derived extracellular vesicles *in vitro*. To this end, primary mouse bone marrow–derived macrophages (mBMMΦs) were polarized toward a pro-inflammatory phenotype using lipopolysaccharide (LPS) and interferon-γ (IFN-γ), followed by treatment with EVs isolated from either naïve or hTERT-immortalized DPSCs. Inflammatory activation of mBMMΦs resulted in a robust induction of classical pro-inflammatory cytokines, including tumor necrosis factor-α (TNFα), interleukin-1β (IL-1β), and interleukin-6 (IL-6), as evidenced by significant increases in both mRNA expression and secreted protein levels (**Figures 3A–F**). Treatment with EVs derived from either naïve DPSCs or hTERT-DPSCs significantly attenuated LPS/IFN-γ–induced inflammatory responses, leading to significant reductions in TNFα, IL-1β, and IL-6 expression at both the transcriptional and protein levels. Importantly, no statistically significant differences were observed between macrophages treated with naïve DPSC-derived EVs and those treated with hTERT-DPSC-derived EVs, indicating that immortalization did not compromise EV-mediated immunomodulatory function.

Collectively, these findings demonstrate that extracellular vesicles derived from immortalized DPSCs retain anti-inflammatory efficacy comparable to EVs from naïve DPSCs, effectively suppressing macrophage inflammatory activation.

### hTERT immortalization preserved EV efficacy across early and mid-passages (P5, P10, and P15), with no detectable decline through passage 15

**Figure 4:**
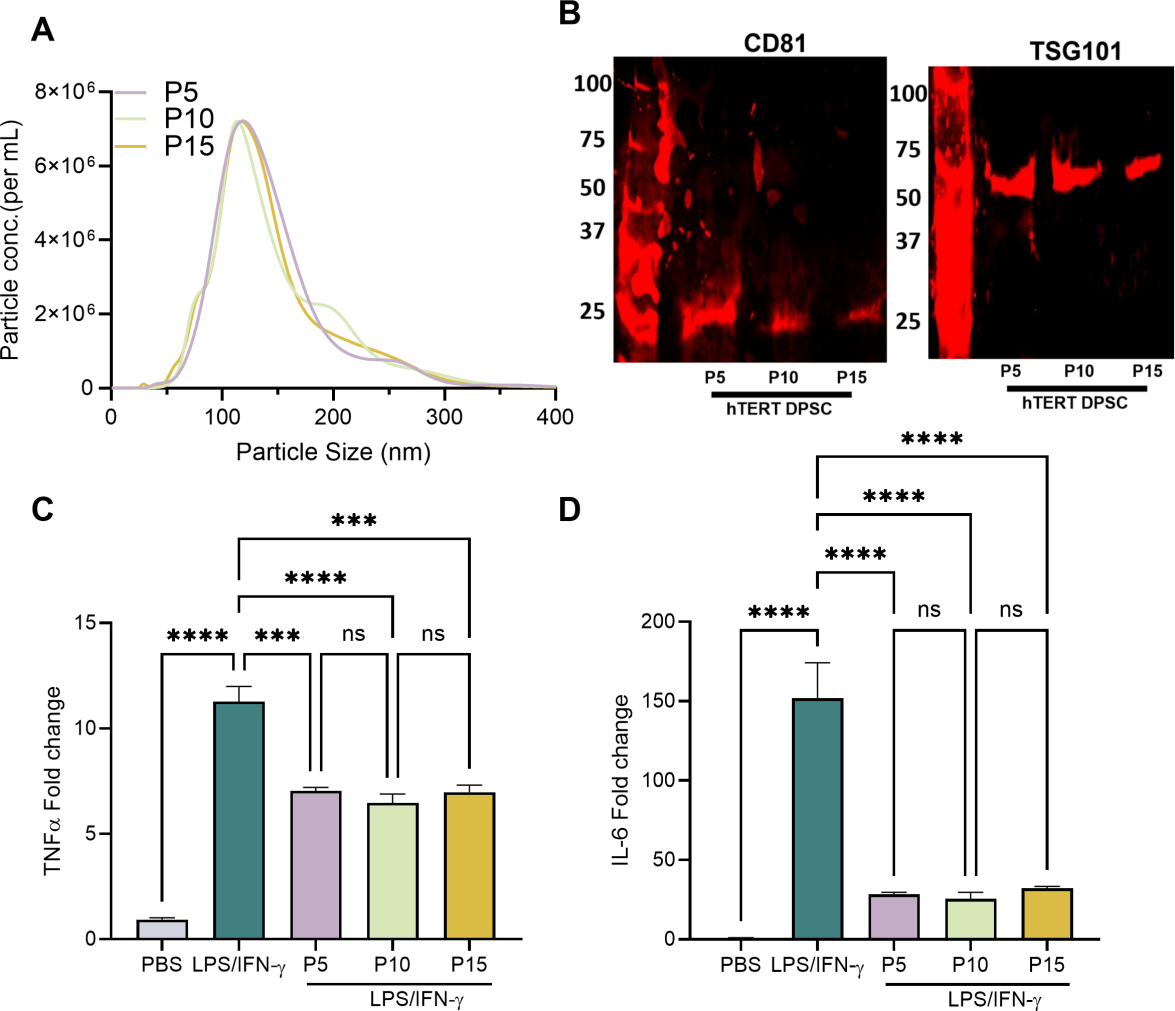
hTERT immortalization preserves EV anti-inflammatory activity across extended passages. EVs were isolated from hTERT-immortalized DPSCs at early (P5), intermediate (P10), and late (P15) passages and evaluated for immunomodulatory activity. **(A)** Representative nanoparticle tracking analysis (NTA) profiles showing EV size distribution. **(B)** Immunoblot analysis of EV markers CD81 and TSG101. **(C, D)** Relative expression of inflammatory cytokines in LPS/IFN-γ–activated mBMMΦs treated with EVs from different passages. Data are presented as mean ± SEM (n = 3). Statistical significance was determined by one-way ANOVA followed by Šídák’s multiple-comparison test. ns- not significant, ***p < 0.001, ****p < 0.0001.

Given our prior observations that primary DPSCs undergo replicative senescence and progressively lose functional activity with extended passaging, we examined whether hTERT-mediated immortalization preserves EV bioactivity across prolonged culture expansion. Immortalized DPSCs were maintained in culture up to passage 15, and extracellular vesicles were isolated from conditioned media at early (P5), intermediate (P10), and late (P15) passages. EVs obtained from each passage were normalized and subsequently evaluated for anti-inflammatory activity using primary mouse bone marrow–derived macrophages (mBMMΦs). Nanoparticle tracking analysis (NTA) and Immunoblot revealed no significant differences in EV particle size and EV markers between passage 5, 10 and 15 of hTERT-DPSC-derived EVs (**Figures 4A, B**).

To assess functional potency, mBMMΦs were polarized toward a pro-inflammatory phenotype using LPS and IFN-γ, followed by treatment with EVs isolated from hTERT-DPSCs at passages 5, 10, or 15. Polarization macrophages resulted in a robust induction of inflammatory mediators, confirming effective macrophage activation. Treatment with EVs from all three passages significantly attenuated inflammatory responses, as evidenced by comparable reductions in pro-inflammatory cytokine expression across P5-, P10-, and P15-derived EV treatment groups (**Figure 4C, D**).

Importantly, no significant differences in anti-inflammatory efficacy were observed among EVs isolated from early, intermediate, or late passages, indicating that prolonged culture of hTERT-DPSCs does not compromise EV-mediated immunomodulatory activity. These findings demonstrate that hTERT immortalization effectively prevents senescence-associated loss of function and enables sustained production of bioactive EVs with preserved anti-inflammatory efficacy through at least passage 15. The absence of changes in EV properties and function suggests minimal off-target effects of transduction.

### *In vivo* evaluation in an acute lung injury model demonstrates that hTERT immortalization does not compromise the therapeutic activity of DPSC-derived EVs

Our *in vitro* studies established that hTERT-mediated immortalization of dental pulp stem cells does not compromise the anti-inflammatory properties of their extracellular vesicles, with EVs maintaining robust suppression of pro-inflammatory macrophage responses. To extend these findings and confirm functional preservation *in vivo*, we evaluated the therapeutic efficacy of EVs derived from naïve and immortalized DPSCs using a murine acute lung injury (ALI) model, a well-established system for assessing innate immune–driven inflammatory responses.

**Figure 5:**
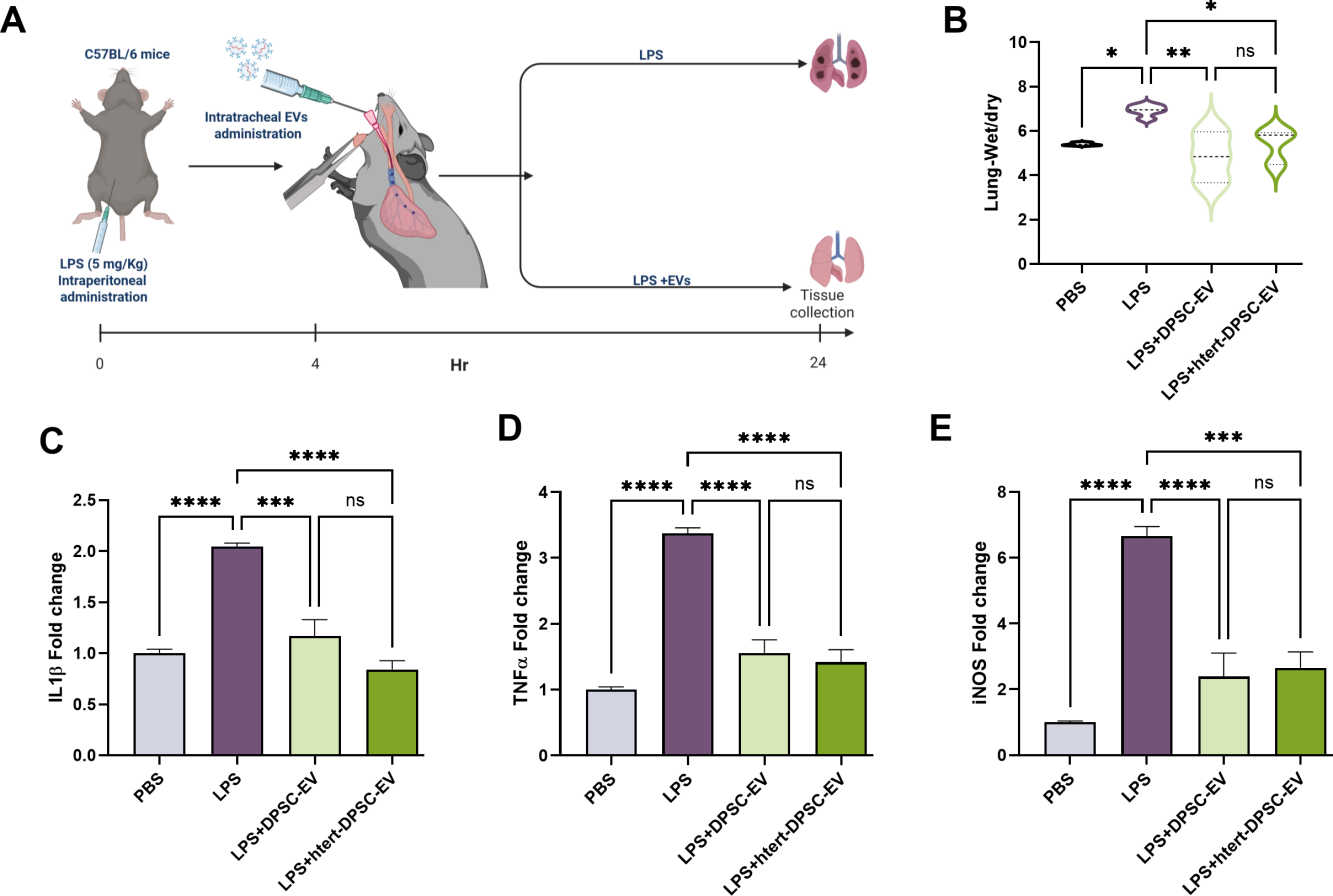
EVs from naïve and hTERT-immortalized DPSCs reduce lung injury in a murine model of acute lung inflammation. Acute lung injury was induced in mice by intraperitoneal LPS administration, followed by intratracheal treatment with EVs derived from naïve or hTERT-immortalized DPSCs. **(A)** Study design schematic outlining the treatment timeline **(B)** Lung wet-to-dry weight ratio as a measure of pulmonary edema. **(C–E)** Relative expression of inflammatory genes in lung tissue assessed by qRT-PCR. Data are presented as mean ± SEM (n = 4). Statistical significance was determined by one-way ANOVA followed by Šídák’s multiple-comparison test. ns- not significant, *p < 0.05, **p < 0.01, ***p < 0.001, ****p < 0.0001.

Acute lung injury was induced by intraperitoneal administration of sub-lethal dose of lipopolysaccharide (LPS), and mice were treated 4 hours after LPS challenge with EVs isolated from either naïve DPSCs or hTERT-immortalized DPSCs, with PBS serving as a control (**Figure 5A**). EVs were administered intratracheally. Lung tissues were collected 24 hours after LPS exposure to assess inflammatory injury. Quantitative analysis revealed a significant reduction in pulmonary edema in mice treated with EVs from both naïve and immortalized DPSCs compared with PBS-treated controls (**Figure 5B**). Importantly, the extent of edema reduction was comparable between the two EV-treated groups, indicating that hTERT immortalization does not impair the ability of DPSC-derived EVs to ameliorate LPS-induced lung injury.

**Figure 6:**
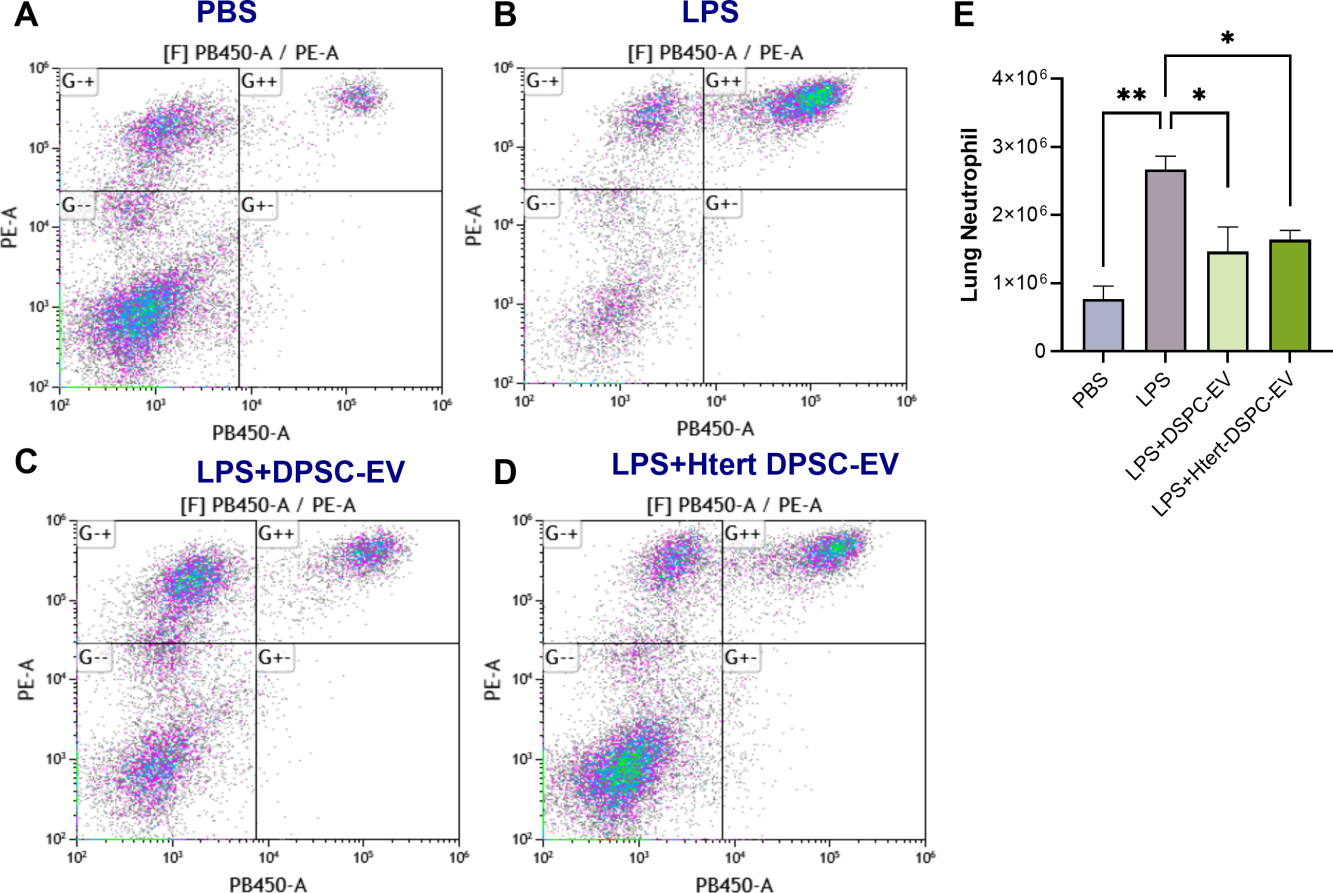
Flow cytometric analysis of lung neutrophil infiltration. **(A–D)** Representative flow cytometry histograms of Ly6G expression on lung neutrophils (CD45^+^CD11b^+^Ly6G^+^), gated on live CD45^+^ cells. **(D)** Flow cytometric quantification of lung neutrophils (CD45^+^CD11b^+^Ly6G^+^). Data are presented as mean ± SEM (n = 4). Statistical significance was determined by one-way ANOVA followed by Šídák’s multiple-comparison test. *p < 0.05, **p < 0.01.

In agreement with these observations, analysis of lung inflammatory gene expression demonstrated similar suppression of pro-inflammatory cytokines in mice treated with naïve or hTERT-DPSC-derived EVs, further confirming preserved immunomodulatory activity following immortalization **Figures 5C–E**. Given the critical role of neutrophil recruitment in ALI pathogenesis, we next assessed whether immortalized DPSC-derived EVs retain the capacity to modulate neutrophil accumulation *in vivo*. Flow cytometric profiling of lung cell populations revealed a pronounced reduction in neutrophil infiltration in both EV-treated groups relative to PBS controls, with no significant difference observed between naïve and hTERT-DPSC EV treatments **Figures 6A–E**.

To determine whether this preservation of EV function extends beyond DPSCs, parallel experiments were performed using EVs derived from naïve and hTERT-immortalized bone marrow derived BM-MSCs. Consistent with the DPSC findings, EVs from naïve and immortalized BM-MSCs exhibited comparable anti-inflammatory efficacy invitro and ALI model (**Supplementary Figures 1H–K**).

Collectively, these *in vivo* data corroborate our *in vitro* results and demonstrate that hTERT-mediated immortalization does not compromise the anti-inflammatory activity of stem cell–derived EVs. Immortalized DPSC-derived EVs effectively reduce pulmonary edema, suppress inflammatory gene expression, and limit neutrophil infiltration in acute lung injury.

## Discussion

Extracellular vesicles derived from mesenchymal stem cells have emerged as a promising cell-free therapeutic platform due to their capacity to modulate inflammation and promote tissue repair while avoiding many of the safety concerns associated with live cell transplantation. Among these, dental pulp stem cell (DPSC)–derived EVs have gained particular interest because of their strong immunomodulatory properties and neural crest origin (32–34). However, a critical barrier to the manufacturing-relevant platform of DPSC-derived EVs is the limited replicative lifespan of primary DPSCs and the associated decline in EV bioactivity with increasing passage number (19, 20). In the present study, we demonstrate that hTERT-mediated immortalization of DPSCs effectively overcomes senescence-associated functional decline while preserving EV physicochemical characteristics, molecular cargo, and anti-inflammatory efficacy both *in vitro* and *in vivo*. The study was designed to evaluate suppression of inflammatory activation and primarily focused on the functional preservation of EV activity rather than detailed mechanistic dissection of EV cargo. Future studies will investigate macrophage polarization, efferocytosis, and cell-specific targeting mechanisms, while comprehensive multi-omics profiling will be required to identify the specific effector molecules responsible for these biological effects.

Our data confirms that hTERT immortalization resulted in a significant increase in telomerase expression without altering EV size distribution, morphology, or expression of canonical EV markers. These findings indicate that telomere stabilization does not disrupt EV biogenesis or vesicle integrity, addressing a key concern regarding the use of immortalized cells for EV production. Preservation of EV uptake by macrophages further supports the conclusion that hTERT expression does not interfere with fundamental EV–recipient cell interactions. Functionally, EVs derived from both naïve and hTERT-immortalized DPSCs exhibited equivalent anti-inflammatory activity in macrophages. Although hTERT expression is associated with cellular immortalization, EVs derived from these cells did not exhibit evidence of pathological signaling *in vivo*. Though detailed cargo and safety profiling will be required for clinical translation. Suppression of TNFα, IL-1β, and IL-6 at both the transcriptional and protein levels underscore the potency of DPSC-EVs in regulating macrophage-driven inflammation. Notably, this immunomodulatory activity was retained in EVs isolated from immortalized DPSCs through at least passage 15. These findings suggest that senescence-associated alterations in EV bioactivity are not an inevitable consequence of prolonged culture per se, but rather reflect telomere-driven cellular aging that can be mitigated through controlled telomerase reactivation.

The preservation of EV function across passages is particularly relevant in light of increasing evidence that EV cargo composition including miRNAs is highly sensitive to cellular stress, senescence, and replicative exhaustion (35, 36). Our observation that miRNA profiles remain largely unchanged between naïve and immortalized DPSC-EVs supports the notion that hTERT immortalization stabilizes the cellular state and prevents senescence-associated reprogramming of EV cargo. This stabilization is likely a key mechanism underlying the sustained anti-inflammatory efficacy observed across extended passages.

The translational relevance of these findings is further strengthened by our *in vivo* validation using a murine acute lung injury (ALI) model, a clinically relevant model characterized by excessive innate immune activation, cytokine production, and neutrophil infiltration (37–39). Both naïve and hTERT-DPSC-derived EVs significantly reduced pulmonary edema, suppressed pro-inflammatory gene expression, and limited neutrophil accumulation in the lungs following LPS challenge. The comparable efficacy of EVs from naïve and immortalized DPSCs demonstrates that hTERT-mediated immortalization does not compromise therapeutic performance in a complex inflammatory environment. Importantly, neutrophil suppression—an essential determinant of ALI severity and outcome—highlights the capacity of DPSC-EVs to modulate both macrophage-driven cytokine signaling and downstream innate immune cell recruitment. The current study focuses on early inflammatory responses (24 h), and therefore does not capture the resolution phase of lung injury. Future studies will extend these findings to later time points (48–72 h) to evaluate resolution-associated pathways including macrophage efferocytosis and IL-10–associated signaling.

Our findings are further reinforced by parallel experiments using EVs derived from naïve and immortalized human mesenchymal stem cells (BM-MSCs), which yielded similar outcomes. This suggests that the preservation of EV bioactivity following hTERT immortalization may represent a generalizable strategy across mesenchymal stem cell sources, rather than a phenomenon unique to DPSCs. Such generalizability is particularly important for establishing standardized, scalable EV manufacturing pipelines. These findings suggest potential applicability across MSC sources, but require validation in additional stem cell types.

From a translational and manufacturing perspective, these results address a major bottleneck in EV-based therapeutic development. The reliance on early-passage primary stem cells severely limits EV yield, batch consistency, and scalability (19, 23). hTERT-immortalized DPSCs offer a renewable and stable EV-producing platform that maintains functional integrity across extended expansion, thereby enabling large-scale, reproducible EV production. In conclusion, this study demonstrates that hTERT-mediated immortalization of DPSCs preserves EV biogenesis, molecular composition, and immunomodulatory function while preventing senescence-associated loss of efficacy. Immortalized DPSC-derived EVs retain potent anti-inflammatory activity *in vitro* and *in vivo*, reduce inflammatory injury, and regulate innate immune cell recruitment. These findings establish hTERT-immortalized DPSCs as a robust and scalable EV source and support their further development as a clinically viable, cell-free therapeutic platform for inflammatory and regenerative indications.

## Methods

### Immortalization of DPSCs (hTERT or iDPSCs)

To generate immortalized DPSCs, early-passage dental pulp stem cells (DPSCs; passage 2) were immortalized through lentiviral transduction with a vector encoding human telomerase reverse transcriptase (hTERT- addgene #176062) driven by a constitutive promoter (CMV) and containing a hygromycin resistance cassette (40). Cells were seeded 24 h prior to transduction and infected with the lentivirus at a multiplicity of infection (MOI) of 200 determined based on optimization experiments. Antibiotic selection was initiated 72 h post-transduction using hygromycin (0.4 mg/ml) and maintained for 5–10 days until non-transduced control cells were eliminated. Successful hTERT expression in the selected cell population was verified by RT-qPCR.

### Cell culture

Human BM-MSC/DPSCs and were procured from Lonza (Catalog: PT-5025) and hTERT-BM-MSC and hTERT-DPSC cells generated in our laboratory were cultured in alpha-MEM containing 20% fetal bovine serum (Gibco), 1% L-Glutamine (Gibco) and 1% antibiotic-antimycotic solution (Gibco). Mouse bone marrow derived macrophages (mBMMs) were isolated from 8-week C57BL/6J mice. Mouse bone marrow cells were cultured with mouse M-CSF (20 ng/ml) for 5 days to obtain myeloid cells differentiated *in vitro* as MΦs (10% FBS/DMEM). For polarizing macrophage towards inflammatory, mBMMs were stimulated with lipopolysaccharides (100 ng/ml, Sigma) with Interferon gamma (50 ng/ml, Peprotech) for 24 hours (41, 42).

### Extracellular vesicles isolation

EVs were isolated from the regular DPSC, BM-MSC and hTERT-DPSC, hTERT BM-MSC according to established protocols (43, 44). Briefly, Cells were cultured under serum free condition for 48 hours. The culture medium was harvested and centrifugation (1500 ×*g)* for 15 min to remove cell debris. The medium was used to isolate EVs according to ExoQuick TC isolation reagent (System Bio- sciences).

### Characterization of extracellular vesicles

Extracellular vesicles were collected from conditioned serum-free culture media using the ExoQuick-TC precipitation method (System Biosciences) according to the manufacturer’s instructions. Particle size distribution and vesicle concentration were quantified by nanoparticle tracking analysis (NTA) using a NanoSight NS300 system (Malvern Instruments).

For protein marker analysis, EV pellets were lysed in RIPA buffer, and equivalent amounts of protein were resolved on 4–20% gradient Mini-PROTEAN TGX precast gels (Bio-Rad). Immunoblotting was performed to detect established EV-associated markers, including CD81 (Abcam, ab109201) and TSG101 (Abcam, ab125011).

The morphology and ultrastructural features of isolated EVs were confirmed by transmission electron microscopy (TEM). Briefly, EV suspensions (5 μL) were fixed in 2% paraformaldehyde and deposited onto formvar/carbon-coated copper grids. After incubation for 30 min at room temperature, samples were post-fixed with 1% glutaraldehyde, followed by extensive washing with distilled water. Grids were negatively stained using 2% uranyl oxalate and subsequently embedded in a uranyl acetate–methyl cellulose mixture. EVs were visualized using a JEOL JEM-1011 transmission electron microscope.

### Quantitative and qualitative assessment of EV uptake

For quantitative uptake analysis, EVs were fluorescently labeled with CellTracker™ Green CMFDA dye (Thermo Fisher Scientific). Murine bone marrow–derived macrophages (mBMMs) were seeded into 96-well plates at 1 × 10^4^ cells per well and exposed to increasing doses of labeled EVs for 2 h at 37 °C. Cells were then washed thoroughly with PBS, and intracellular fluorescence was quantified using a BioTek Cytation microplate reader.

For qualitative uptake studies, mBMMs (5 × 10^4^ cells) were plated onto sterile coverslips in 12-well plates. Labeled EVs (1.8 × 10^9^ particles per well) were added and incubated for 2 h. Following incubation, cells were washed with PBS, fixed with 4% paraformaldehyde, permeabilized, and stained with Alexa Fluor^®^ 568–conjugated phalloidin (Invitrogen) to visualize the actin cytoskeleton. Coverslips were mounted using DAPI-containing mounting medium (Vector Laboratories), and images were acquired using a Zeiss LSM 710 Meta confocal microscope.

### Multilineage differentiation, histological staining, and gene expression analysis

To evaluate the multilineage differentiation capacity of stem cells, osteogenic, chondrogenic, and adipogenic differentiation was induced using lineage-specific culture conditions (44). Cells were maintained in differentiation media for four weeks, with medium changes performed every 3 days for 2 weeks. Osteogenic differentiation was induced by culturing cells in α-minimum essential medium (α-MEM) supplemented with ascorbic acid (100 μg/mL), β-glycerophosphate (10 mM), and dexamethasone (10 nM). Following the induction period, mineralized matrix formation was assessed by Alizarin Red S staining. Chondrogenic differentiation was performed by culturing cells in α-MEM basal medium supplemented with dexamethasone (1 μM), ascorbate-2-phosphate (50 μg/mL), insulin–transferrin–selenium (ITS) supplement (1%), fetal bovine serum (1%), and transforming growth factor-β1 (TGF-β1; 10 ng/mL). After two weeks of induction, extracellular matrix proteoglycan production was assessed using Safranin staining. Adipogenic differentiation was induced by culturing cells in growth medium supplemented with insulin (10 μg/mL), isobutyl-1-methylxanthine (IBMX; 500 μM), indomethacin (100 μM), and dexamethasone (1 μM). Lipid droplet accumulation was visualized by Oil Red O staining. For gene expression analysis, total RNA was isolated from differentiated and control cells using a commercial RNA extraction kit according to the manufacturer’s instructions and Lineage-specific marker genes were analyzed to confirm differentiation.

ELISA for cytokine measurements: mouse *invitro differentiated* macrophages (mBMMs) were pretreated with EVs from regular DPSC/BM-MSC and hTERT-DPSC/BM-MSC with LPS/IFN-γ for 24 hours. Conditioned media was collected and cytokine levels of IL-1β, IL-6, TNF-α, were measured using DuoSet ELISA (enzyme-linked immunosorbent assay) kits (R&D Systems, MN).

***Real-time RT-PCR:*** RNA was extracted using the RNeasy^®^ Mini Kit (Qiagen) and then reverse transcribed to cDNA using the RevertAid RT Reverse Transcription Kit (Thermo Scientific) for subsequent quantitative real-time PCR (qRT-PCR) analysis. qRT-PCR was conducted using mouse primers obtained from Integrated DNA Technologies for Interleukin-1 beta (IL-1β), Tumor Necrosis Factor alpha (TNF-α), Interleukin-6 (IL-6) along with the SYBR green gene expression master mix (Applied Biosystems). The data are presented as fold changes in RNA levels compared to the control treatment, calculated using the 2−ΔΔCt method.

### Murine model of LPS-induced acute lung injury

All animal studies were approved by UIC Animal Care and Use Committee (protocol # 2024-053). After 7 days of acclimatization, 8-10-weeks-old male C57BL/6J mice (Jackson Laboratory) were divided into six groups; a) Control b) LPS (5 mg/kg-ALI Model) c) LPS + DPSC EV d) LPS + hTERT-DPSC EV e) LPS + BM-MSC EV f) LPS + hTERT-BM-MSC EV, (n=8). Acute lung injury was induced by intraperitoneal injection of lipopolysaccharide (LPS; 5 mg/kg body weight). Four hours after LPS administration, mice in the treatment groups received a single intratracheal extracellular vesicles (1 × 10^8^ particles suspended in 50 µL sterile PBS). Control animals received vehicle alone. Animals were monitored throughout the experimental period for signs of distress. Twenty-four hours following LPS, mice were euthanized humanely according to institutional guidelines. Lung tissues and relevant biological samples were harvested immediately for downstream analyses (45–47).

### Wet/dry ratio for lung edema

PBS-perfused lungs from mice were excised at defined time points following LPS-induced injury, weighed to determine the wet weight. The lungs were then dried in an 80°C oven for 3 days and reweighed to determine the dry weight. The wet-to-dry ratio was calculated as a measure of lung edema (38, 48).

### Preparation of mouse lung single-cell suspensions for flow cytometry

Lungs from euthanized mice were perfused with 10 mL of phosphate-buffered saline (PBS) via the right ventricle, excised, and finely minced using scissors. Lung tissues were enzymatically digested in collagenase A (1 mg/mL; Millipore-Sigma) in a shaking water bath at 37 °C for 45–60 min. Following digestion, tissues were mechanically dissociated by passage through a 18-gauge needle and filtered through a 70-µm nylon mesh to obtain a single-cell suspension. Red blood cells were lysed using 1X RBC lysis buffer according to manufacture protocol (38). For flow cytometric analysis, lung single-cell suspensions were resuspended in FACS buffer (PBS + 1%BSA+2mM EDTA, BD Biosciences) and incubated with Fc-blocking antibody (TruStain; BioLegend) for 10 min to minimize nonspecific antibody binding. Cells were then stained with fluorophore-conjugated antibodies against mouse CD45 (30-F11, biolegend), CD11b (M1/70, Biolegend), and Ly6G (1A8, Biolegend). Neutrophils were identified CD45+Ly6G+CD11b+ and Data acquisition was performed using Gallios and CytoFLEX S flow cytometers (Beckman Coulter), and data were analyzed using Kaluza Analysis software v2.1 (Beckman Coulter) and FlowJo.

### Statistical analysis:

Statistical analyses were performed using GraphPad Prism 10. Differences among multiple groups were evaluated by one-way ANOVA followed by Šídák’s *post hoc* tests for multiple comparisons. All data are presented as mean ± SEM, and statistical significance was defined as *p* < 0.05.

## Data Availability

The original contributions presented in the study are included in the article/**Supplementary Material**. Further inquiries can be directed to the corresponding authors.

## Supplementary material

Supplementary Figure 1 Preservation of EV immunomodulatory activity following hTERT immortalization extends to BM-MSCs. EVs derived from naïve and hTERT-immortalized human mesenchymal stem cells (BM-MSCs) were evaluated in vitro and in vivo. **(A)** Relative hTERT mRNA expression in naïve and immortalized BM-MSCs assessed by qRT-PCR. **(B)** Quantitative uptake of fluorescently labeled EVs by mouse bone marrow–derived macrophages (mBMMϕs) measured after 2 h incubation. **(C)** Representative confocal microscopy images showing intracellular localization of EVs (green) within macrophages, with actin cytoskeleton (red) and nuclei (blue). **(D)** Representative nanoparticle tracking analysis (NTA) profiles showing EV size distribution. **(E–G)** Relative mRNA expression of pro-inflammatory cytokines TNF-α, IL-1β, and IL-6 assessed by qRT-PCR. miRs expression in EVs isolated from naïve and hTERT-DPSC. **(H–K)** Lung wet-to-dry weight ratio and relative expression of inflammatory genes in lung tissue assessed by qRT-PCR. Data are representative of at least three independent experiments. Data are presented as mean ± SEM (n = 4). Statistical significance was determined by one-way ANOVA followed by Šídák’s multiple-comparison test. ns- not significant, *p < 0.05, **p < 0.01, ***p < 0.001, ****p < 0.0001.

Supplementary Figure 2 Gating strategy used for flow cytometric analysis.
