# Supplementary figures and images for: Telomerase-mediated immortalization preserves the anti-inflammatory activity of dental pulp stem cell extracellular vesicles
Source: Front Immunol. 2026 Jun 10;17:1833834. doi: 10.3389/fimmu.2026.1833834 (PMC13290532; doi:10.3389/fimmu.2026.1833834)

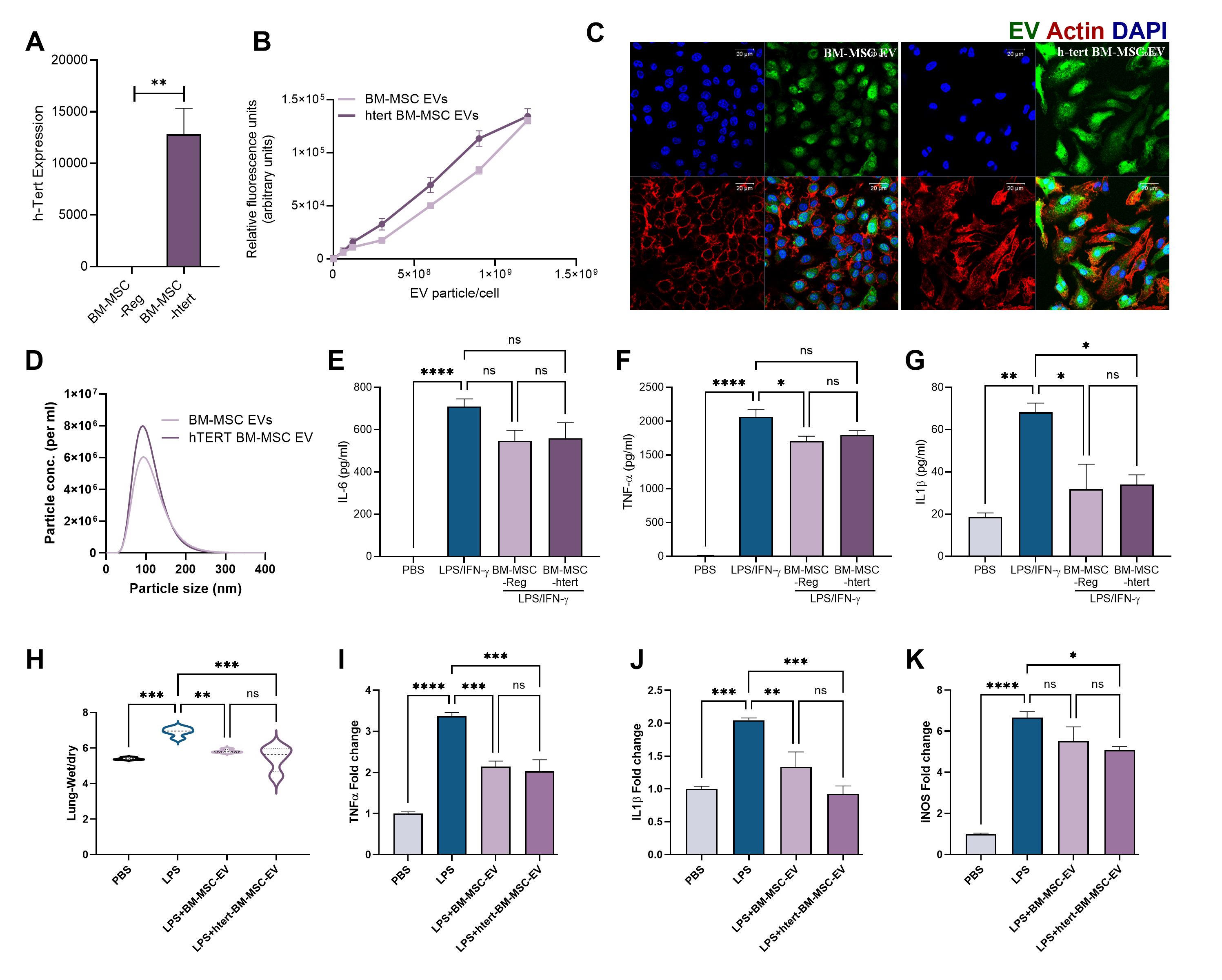

Supplement: Supplementary Figure 1 — Preservation of EV immunomodulatory activity following hTERT immortalization extends to BM-MSCs. EVs derived from naïve and hTERT-immortalized human mesenchymal stem cells (BM-MSCs) were evaluated in vitro and in vivo. (A) Relative hTERT mRNA expression in naïve and immortalized BM-MSCs assessed by qRT-PCR. (B) Quantitative uptake of fluorescently labeled EVs by mouse bone marrow–derived macrophages (mBMMϕs) measured after 2 h incubation. (C) Representative confocal microscopy images showing intracellular localization of EVs (green) within macrophages, with actin cytoskeleton (red) and nuclei (blue). (D) Representative nanoparticle tracking analysis (NTA) profiles showing EV size distribution. (E–G) Relative mRNA expression of pro-inflammatory cytokines TNF-α, IL-1β, and IL-6 assessed by qRT-PCR. miRs expression in EVs isolated from naïve and hTERT-DPSC. (H–K) Lung wet-to-dry weight ratio and relative expression of inflammatory genes in lung tissue assessed by qRT-PCR. Data are representative of at least three independent experiments. Data are presented as mean ± SEM (n = 4). Statistical significance was determined by one-way ANOVA followed by Šídák’s multiple-comparison test. ns- not significant, *p < 0.05, **p < 0.01, ***p < 0.001, ****p < 0.0001. [file Image1.tif]

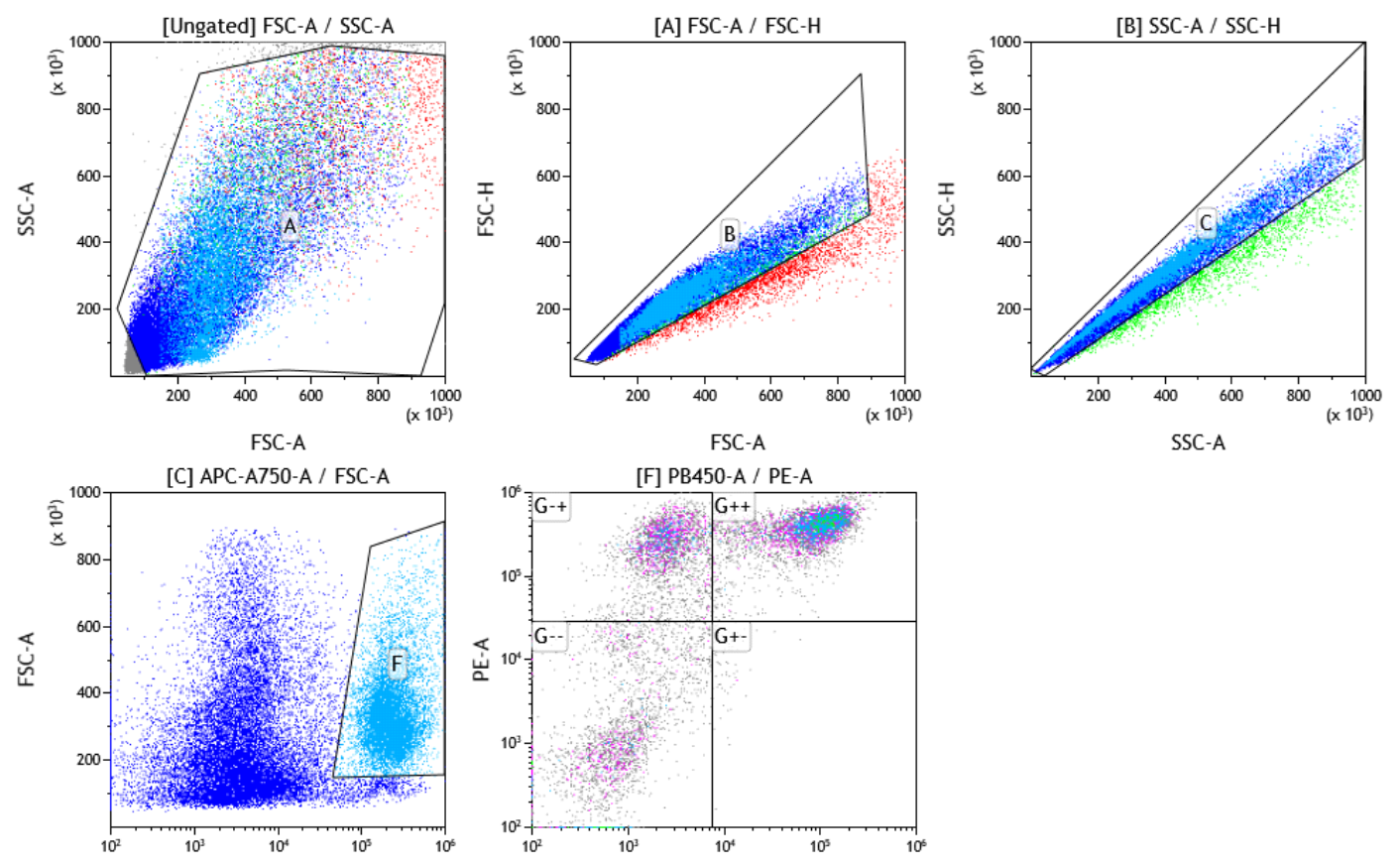

Supplement: Supplementary Figure 2 — Gating strategy used for flow cytometric analysis. [file Image2.tif]
